# Biomechanical finite element analysis of short-implant-supported, 3-unit, fixed CAD/CAM prostheses in the posterior mandible

**DOI:** 10.1186/s40729-022-00404-8

**Published:** 2022-02-11

**Authors:** Lana Zupancic Cepic, Martin Frank, Andreas Reisinger, Dieter Pahr, Werner Zechner, Andreas Schedle

**Affiliations:** 1 Department of Prosthodontics, University Clinic of Dentistry, Medical University of Vienna, Vienna, Austria; 2 Institute of Lightweight Design and Structural Biomechanics, TU Wien, Vienna, Austria; 3 Division Biomechanics, Department of Anatomy and Biomechanics, Karl Landsteiner University of Health Sciences, Krems, Austria; 4 Department of Oral Surgery, University Clinic of Dentistry, Medical University of Vienna, Vienna, Austria; 5 Competence Centre Dental Materials, University Clinic of Dentistry, Medical University of Vienna, Vienna, Austria

**Keywords:** Biomechanics, Finite element analysis, Short dental implants, Fixed implant-supported prostheses, Prosthetic design parameters, Functional load

## Abstract

**Objective:**

To assess the biomechanical effects of different prosthetic/implant configurations and load directions on 3-unit fixed prostheses supported by short dental implants in the posterior mandible using validated 3-D finite element (FE) models.

**Methods:**

Models represented an atrophic mandible, missing the 2nd premolar, 1st and 2nd molars, and rehabilitated with either two short implants (implant length-IL = 8 mm and 4 mm) supporting a 3-unit dental bridge or three short implants (IL = 8 mm, 6 mm and 4 mm) supporting zirconia prosthesis in splinted or single crowns design. Load simulations were performed in ABAQUS (Dassault Systèmes, France) under axial and oblique (30°) force of 100 N to assess the global stiffness and forces within the implant prosthesis. Local stresses within implant/prosthesis system and strain energy density (SED) within surrounding bone were determined and compared between configurations.

**Results:**

The global stiffness was around 1.5 times higher in splinted configurations vs. single crowns, whereby off-axis loading lead to a decrease of 39%. Splinted prostheses exhibited a better stress distribution than single crowns. Local stresses were larger and distributed over a larger area under oblique loads compared to axial load direction. The forces on each implant in the 2-implant-splinted configurations increased by 25% compared to splinted crowns on 3 implants. Loading of un-splinted configurations resulted in increased local SED magnitude.

**Conclusion:**

Splinting of adjacent short implants in posterior mandible by the prosthetic restoration has a profound effect on the magnitude and distribution of the local stress peaks in peri-implant regions. Replacing each missing tooth with an implant is recommended, whenever bone supply and costs permit.

## Background

The biomechanical conditions of dental implants differ from those of natural teeth during functional loading. Occlusal forces are directly transferred to the bone because of the missing periodontal ligament, which would provide a mechano-receptive feedback and a shock-absorbing function of the opposing dentition [1, 2]. Therefore, dental implants exhibit low tactile sensitivity and low proprioceptive motion feedback [2, 3]. As such, implants are unable to deliver feedback or adjust to occlusal overload, which might cause biological and mechanical complications in oral implant rehabilitations [4]. Contributing factors to overload include the pristine bone quality, implant-associated factors (e.g., number, length, type of connection, distribution and inclination), prosthetic design (e.g., static and dynamic occlusal schemes, premature contacts, cantilevers, splinting and crown-to-implant ratio) as well as behavioural habits of the patient (e.g., clenching and bruxism) [5, 6]. Hence, the clinical success and longevity of dental implant supported restorations requires biomechanically adapted implant/restoration loading.

Implant-supported fixed partial dentures (FPD) are widely used as a predictable treatment option for partially edentulous patients [7]. However, the surgical feasibility of implant insertion is limited in some cases. Especially in the posterior regions of the jaws, tooth extractions may lead to significant bone resorption resulting in an insufficient bone volume for the insertion of implants with a standard length of 10 mm or more. In borderline situations short dental implants (≤ 8 mm) have been proposed as an alternative to bone augmentation procedures [8, 9] exhibiting encouraging short-term survival rates [10, 11]. From a biomechanical point of view, however, literature indicates that short implants offer a reduced overall contact area between bone and implants and increase the crown-to-implant ratio [12], which might cause biomechanical complications due to stress accumulation, especially under oblique loads [9, 13]. The concept of splinted prostheses has been suggested to improve stress distribution on the prosthesis, implants, and peri-implant tissues [14, 15]. However, a recent meta-analysis concluded that splinted short implants do not exhibit superior performance in survival rate, marginal bone maintenance and prevention of mechanical complications compared with single-unit prosthesis [16]. In general, the influence of overloading factors on short implant longevity is still inconclusive in the literature. In this sense, we seek to reveal which biomechanics are more favourable for a typical clinical setting in the posterior jaw with implant-supported 3-unit fixed partial dentures (FPD) with short (≤ 8 mm) and extra-short (< 6 mm) implants.

Direct clinical evaluation of the biomechanical aspect of implant treatments would be an optimal setting, however, the difficulties in utmost objectively assessing or quantifying the level of osseointegration and the stability of the implant as well as the potential ethical issues, do make it, in fact, almost impossible [17]. To overcome these limitations, in silico tests such as finite element analysis (FEA), which has become even more sophisticated over time, have been introduced into implant dentistry to analyse the stress distribution in dental implant systems (prosthetic components, implant, and surrounding bone) [18]. FEA uses computational models to simulate and investigate mechanical problems [19] by discretisation of a sample geometry with a mesh of elements and calculation of local forces and displacements using mathematical functions [17]. This method has also been used for the prediction of how a part or assembly behaves under given conditions [20]. The results of a simulation are usually depicted via a colour scale that shows for example the surface pressure or internal stress distribution of an object. However, the accuracy of FEA may be influenced by simplification of the geometry of the bone or implant system, boundary conditions and material properties [21]. Therefore, model validation is an important procedure for the confidence to accurately predict mechanical phenomena, especially when it seems to have clinical implications, and should involve a quantitative comparison of the model outputs against experimental data [22].

The aim of this study was to evaluate the stress distribution in 3-unit fixed partial dentures in the posterior region of an atrophic mandible supported by 3 or 2 short dental implants using validated 3D simulation models and to determine the biomechanical effects of crown splinting and load directions. The null hypotheses were as follows:

(i) splinted and non-splinted crowns exhibit similar stress distribution on implant/prosthesis systems and surrounding bone and
(ii) different implant lengths and loading show no differences in local stress accumulation.

## Materials and methods

### Experimental design

**Fig. 1:**
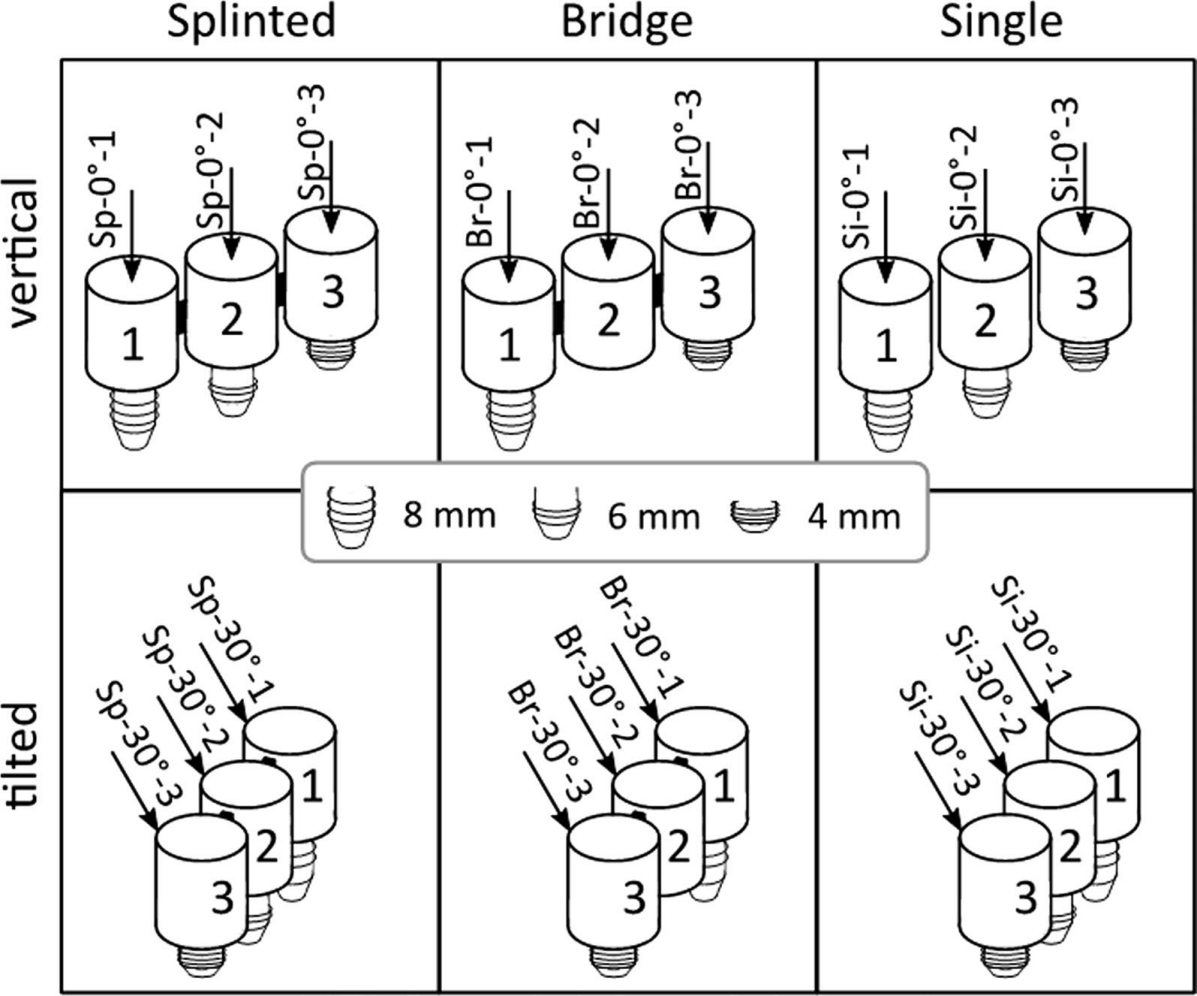
Overview of test configurations. Left: splinted (Sp) crowns on 3 implants. Centre: 3-unit bridge (Br) supported by 2 outer implants with the middle implant missing. Right: Single (Si) crowns on 3 implants. Each crown/unit is loaded separately: 2nd premolar (1), 1st molar (2), and 2nd molar (3). All loads (arrows) are applied vertically (top column, 0°) or tilted at 30° (from buccal to lingual, bottom column). Implant length sequence: 8 mm—6 mm—4 mm from 2nd premolar to 2nd molar

This study involves the establishment of FE-models of edentulous posterior mandibular sites with alveolar bone resorption rehabilitated by short dental implants supporting 3-unit, screw-retained prostheses for biomechanical evaluation of the following configurational variables: prosthesis design (splinted crowns, bridge, or single crowns), implant length (8, 6, and 4 mm), implant number (3 or 2), and loading direction (axial and oblique), illustrated in Fig. 1. The study design follows the EQUATOR guidelines for strengthening the reporting of empirical simulation studies (STRESS) [23].

For this purpose, three implants (Regular Neck, SLActive^®^, Ø4.1 mm, Institut Straumann AG, Basel, Switzerland) in the length sequence 8 mm–6 mm–4 mm from mesial to distal (according to bone supply), were placed into an atrophic posterior mandibular bone sample from the right hemi-arch of a fresh human cadaver (ethical approval Nr. 2235/2019) to replace the 2nd premolar, 1st molar, and 2nd molar according to the manufacturer’s protocol. The implant positions were digitised using an intraoral scanner (TRIOS^®^ 3, 3Shape, Copenhagen, Denmark) for the CAD/CAM fabrication of screw-fixed, monolithic zirconia restorations, simultaneously milled with abutments in splinted and non-splinted (e.g., 3 single crowns) design. To simulate the 2-implant configurations where the middle implant is missing (e.g., 3-units bridge), the connection of the prosthodontic frame to the underlying middle implant was removed. With a crown height (CH) of 11 mm, the crown-to-implant ratio (C/I) obtained for the respective implant length was 1.4, 1.8, and 2.7, respectively (Table 1).

**Table 1:** Respective descriptions of the implants and crowns

	Second premolar	First molar	Second molar
CH	11 mm	11 mm	11 mm
IL	8 mm	6 mm	4 mm
C/I	1.4	1.8	2.7

*CH* crown height, *IL* implant length, *C/I* crown-to-implant ratio

The mandibular bony specimen and all prosthetic configurations were tested mechanically in a static manner under axial and oblique loads in a thorough experimental set-up to validate the numerical simulation findings in a previous study [24].

### Mechanical testing

The rehabilitated mandibular sample was embedded in polymethyl methacrylate (PMMA) and mounted into an aluminium block clamped onto the tilt table of the test rig of a Zwick Z030 test machine (ZwickRoell GmbH, Ulm, Germany), equipped with an optical deformation system (ARAMIS, GOM GmbH, Braunschweig, Germany) to measure the global stiffness and deflection of the bone/implant/prosthesis system from the recorded unloading curves. The force of 100 N was applied in the region of the fossa of each crown vertically and at 30° to the long axis of the assembly (from buccal to lingual) by a piston which either directly loaded the occlusal surface (in case of off-set loading) or by interposing a ball in the centre of each occlusal area to maintain a reliable contact surface with the piston in case of vertical loading.

### Modelling

The FE-model consists of four parts: crown, implant, bone and embedding. Prior to implantation, a microtomography (µCT) scan of the native mandible was performed with a µCT100 (Scanco Medical AG, Switzerland) at a nominal resolution of 11 µm (70 kV, 114 µA, 200 ms integration time, Al 0.5 mm filter) to obtain the bone tissue design without any artefacts. The µCT-scan, captured after implantation and fixation of the prosthesis, was used to determine the geometry of the implants. As the prosthesis caused a large artefact area, the µCT data were not usable for the design determination of the crowns. Thus, CAD*-*files (.*stl*) of both, the un-splinted and splinted crowns, were used to acquire the geometry of the prosthesis.

The 3D-data images were processed in medtool 4.3 (Dr. Pahr Ingenieurs e.U., Pfaffstätten, Austria) to subsequently generate meshes for the bone, implant, and crown with tetrahedral, quadratic, solid elements, using an algorithm based on CGAL (*The Computational Geometry Algorithms Library*). Concretely, µCT images were converted to *mhd* file format and rescaled to 8-bit images (grey values 0–255). Sequentially, both scans were segmented to obtain two binary images (single-level threshold) for bone tissue and implants. Segmentation quality was qualitatively verified with overlay plots of the original files in medtool. The *mhd* file of the implants were then added onto the *mhd* file of the mandible*.* The *stl* files of the prosthesis were edited with Hypermesh 2017.2 (Altair Engineering, Troy, USA), and located at the exact position, according to the µCT scan of the implanted mandible. Then, the *shrinkwrap* function was applied to obtain a 3D mesh of the prosthesis (*inp* file). This file was finally added to the matched *mhd* file of the mandible and implants and meshed with CGAL. The embedding was modelled by generating a solid cuboid, matching a surface laser-scan of the actual embedding. Finally, the regular meshes at the surface of the embedding were created and connected to the mesh of the mandible.

Mechanical properties (Young’s modulus of elasticity and Poisson’s ratio) of each simulated material were attributed to the meshes using literature values [25] as indicated in Table 2.

**Table 2:** Material parameters used for FE-analysis

Component	Material	Elasticity modulus (GPa)	Poisson ratio [−]
Mandible	Bone tissue	13.7	0.3
Prosthesis	Zirconia	200	0.31
Screws/implants	Titanium	110	0.3
Embedding	PMMA	1	0.3

In total 2.1 million quadratic tetrahedral elements (C3D10) were created, with a typical element length of 0.1 mm. The models were considered isotropic, homogenous, and linearly elastic.

The bone–implant interface was assumed to be perfectly bonded to simulate complete osseointegration. As µCT images allowed no discrimination between crowns and implants, this interface was identified as bonded interface so that the implants and prosthetic restorations were assumed to behave as a single unit [26, 27]. The interproximal crown of non-splinted models was simulated without contact by deleting the regions of splinting.

**Fig. 2:**
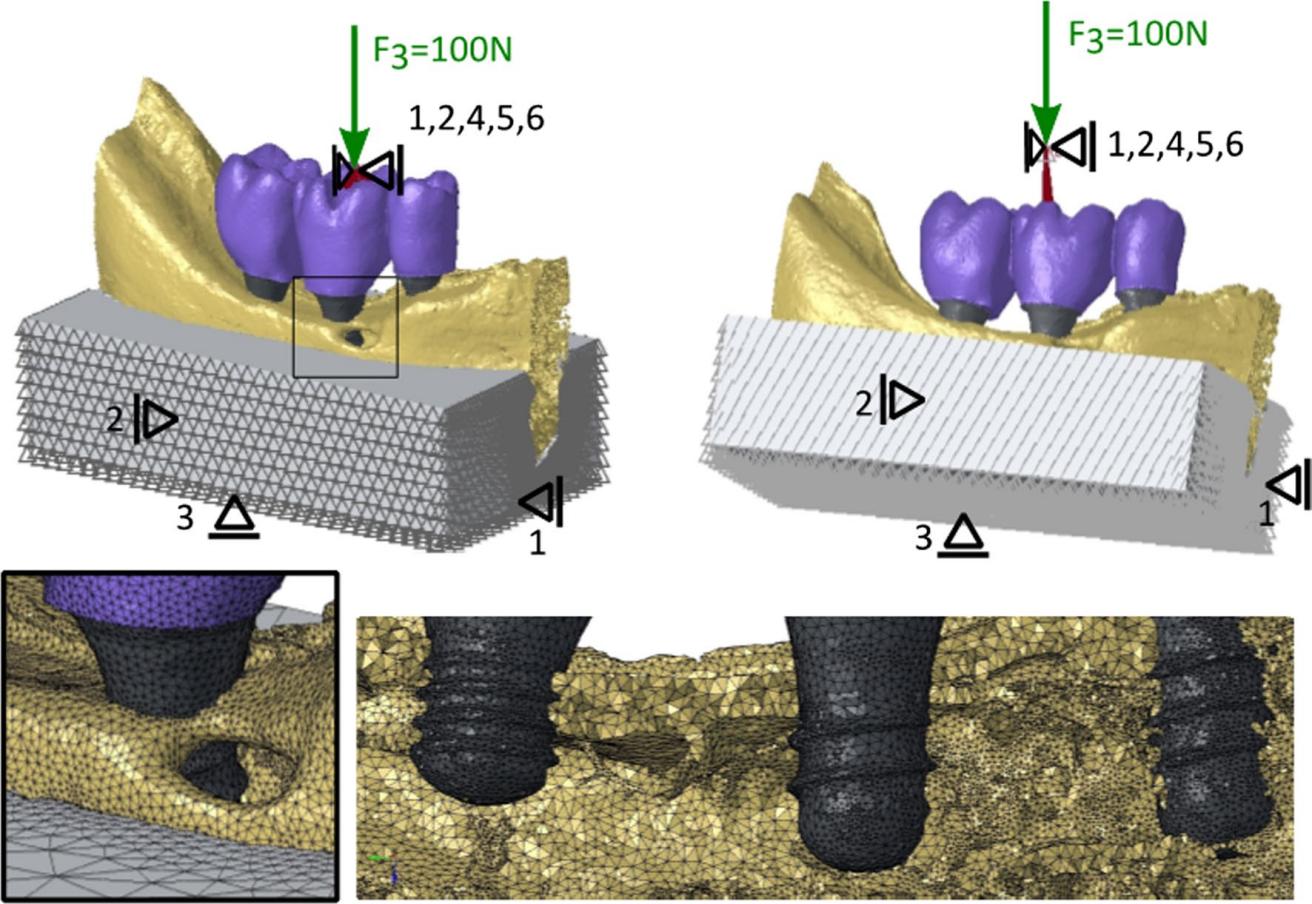
Components, boundary conditions, and mesh density of a study model. An example of the splinted, 3-implant configuration is shown for the loading of the 1st molar. Components of the assembly: prosthetic restoration (violet), implants (dark grey), bone (yellow) and embedding (light grey). The model is aligned vertically (top left) or tilted at 30° (top right). The vertical force of 100 N (green arrow) with DOF 3 is applied on the reference node. The node is constrained in all directions (black support symbols including numbers), except the vertical direction (DOF 3). Kinematic coupling (dark red) is done with all DOFs. The embedding is constrained as indicated. The inset on the left bottom shows the mesh at the boundaries between crown, implant, bone and embedding. The bottom right image illustrates the osseointegration of the implants in the mandible

All mesh elements were feasible for 3-dimensional translation and rotation (*x*, *y*, and *z*-axes). The boundary conditions (Fig. 2) were defined by the six degrees of freedom (DOF) according to measurements of the mechanical experiments. The perspective of the image in Fig. 2 slightly distorted the implants and their diameter therefore appears to be different, although it is 4.1 mm for all implants. A vertical force of 100 N (DOF 3) was applied on the reference node (centre of the ball in mechanical tests), which was related to a rigid kinematic coupling (all DOFs) to the surface known from the experiment. The reference node was constrained in all directions, except the vertical (DOF 3). The embedding was constrained for translational movements (DOF 1, 2 and 3), depending on the surface (as seen in Fig. 2). The computation time took 26 min on average on 4 CPUs (2 × 14 Intel(R) Xeon(R) CPU E5-2697 v3 @ 2.60 GHz, 800 GB memory) for each model.

### FE-analysis

The final FE-models were exported to ABAQUS (V6R2018, Dassault Systèmes, France) for numerical analysis of distribution and concentration of biomechanical stress and deformation within the implant/prosthesis system and surrounding bone. The vertical deflection of the reference node was determined with the Field-Output-reader in medtool. The applied force of 100 N was divided by this deflection to calculate the global stiffness. The von Mises stress was used to assess the stress distribution in the implant/prosthesis/bone ensemble, whereas the strain energy density (SED) was evaluated to get insight into physiological energies present in the bone tissue only. Stresses were analysed with respect to their distribution and magnitude, in comparison to a physiological yield limit of 100 MPa for mandibular bone [28]. Similarly, strain energy density (SED) in bone tissue was also investigated with respect to physiological values of 0.02 MPa [29], marking potential remodelled areas below or above this value.

### FE-validation and verification

The experimental stiffness determined from the FE-analysis was compared with the stiffness from mechanical testing to validate the correlation of the analysis and to determine the factor by which the stiffness is different by the two approaches in a previous study [24]. Further, the numerical simulation was verified by proving the shape of deformation, deformation distribution and stress estimation.

## Results

### Experimental validation

Validation of the FEA was reported in detail in a previous study [24]. In brief, the comparison between experimental and FE-stiffness for the pooled data demonstrated a very high correlation (*R*^2^ = 0.80), but the FEA-stiffness was 7.2 times higher. The factor was highly dependent on the test configuration. However, both evaluation procedures showed the same trend in the difference of the stiffness between the different load cases for each investigated configuration, confirming a similitude of tendencies and proving a qualitative equivalence.

### Global stiffness (FEA)

**Fig. 3:**
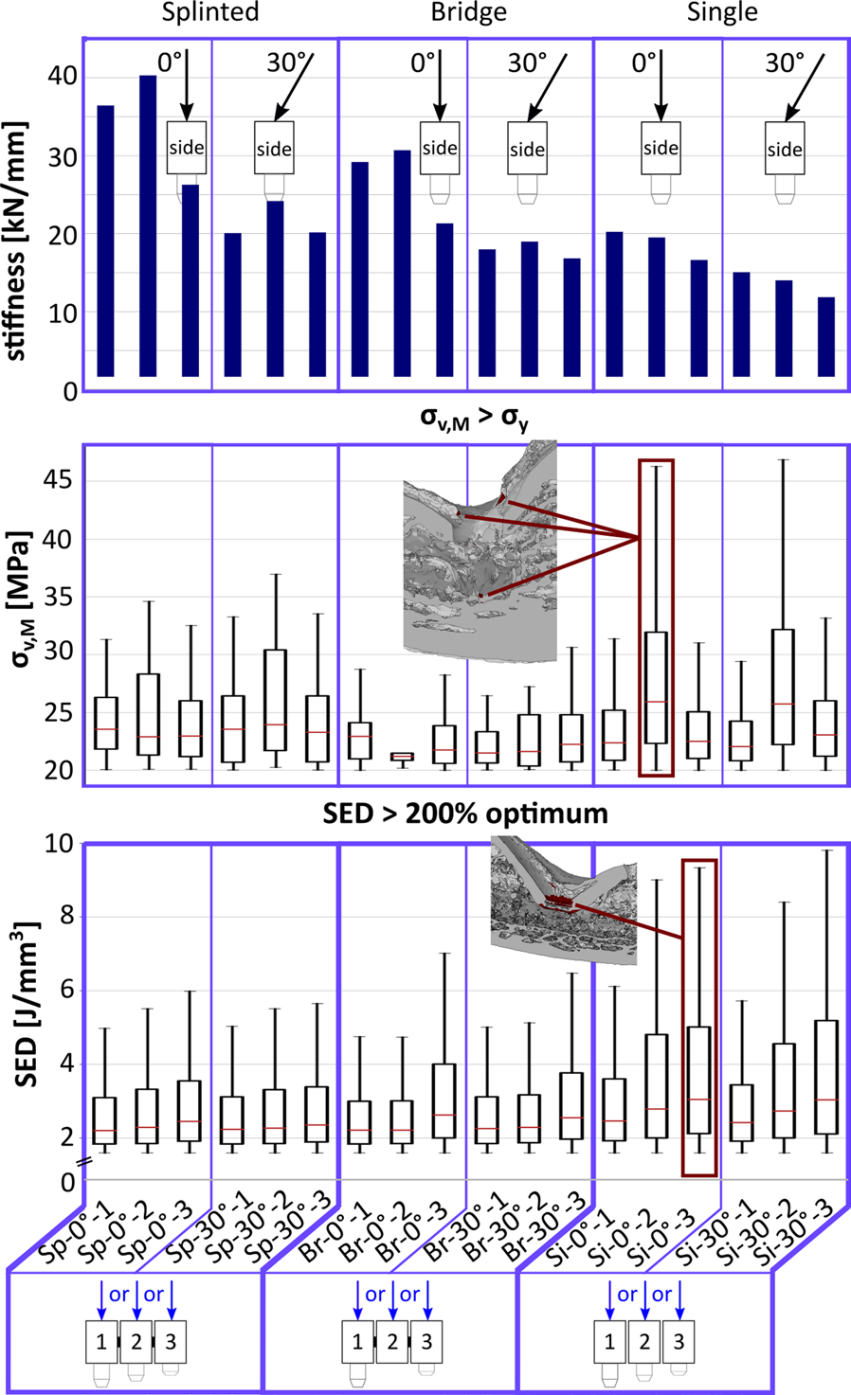
Comparative FE-stiffness, von Mises stress (> yield stress) and SED (> 200% optimum) of different configurations. It is observed that the stiffness values vary depending on the type of prosthetic design and the load direction in each applied position. Note: Boxplots are shown without outliers, for easier comparison across groups. Only values above the yield limit or 200% of the optimum SED are displayed, to demonstrate the fraction of overloaded regions only, corresponding to dark red regions in Figs. 5 and 6. Insets demonstrate bone regions with values above the yield limit or 200% of the optimum SED labelled in dark red for a selected configuration

Figure 3 (top) shows the comparative stiffness for all load configurations obtained from numerical calculation. Obviously, the splinted crowns (Sp & Br) at vertical load (0°) showed the largest stiffness. Hereby, the 3-implant splinted configuration (Sp-0°) was 21% stiffer than the equivalent prosthetic design supported by 2 implants (Br-0°). Further, the stiffness was highest when the vertical load was applied on implant 1 (8 mm) or implant 2 (6 mm) but decreased by 33% when loading the extra-short implant 3 (4 mm) vertically. The off-axis loading (30°) in splinted configurations led to a decrease of stiffness by 39%, but there was almost no difference between the applied load position 1, 2 or 3. The vertical single crowns configuration (Si-0°) resulted in very similar stiffness values as the splinted 2-implant configuration (Br-30°) loaded off-axis. Like in splinted configurations, tilted loading of single crowns led to a decrease of 30% in global stiffness.

### Force distribution on splinted implants

**Fig. 4:**
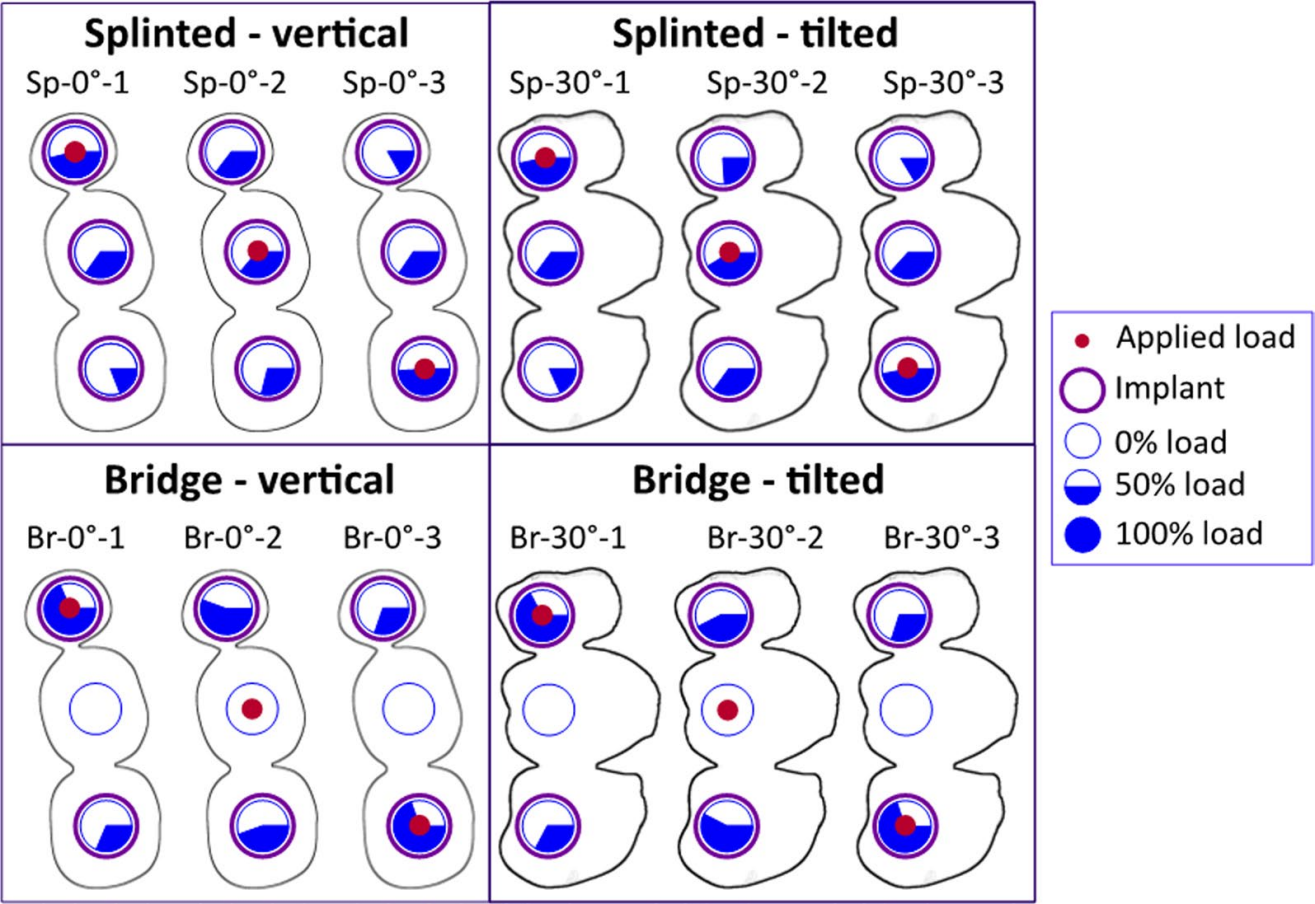
Force distribution on splinted implants. The force effects are higher in the 2-implant configuration (bridge) compared to the loads distributed over 3 splinted implants for both loading directions

Figure 4 illustrates the force distribution on splinted implants (splinted and bridge configurations). Since the applied force was 100 N, the pie charts show both, the relative amount in % as well as the real amount in N. In the Sp configuration, each unit of the prosthesis is supported by an underlying implant, whereby the greatest load (37–55 N) was always at the implant below the crown of the applied force. In general, the force distribution was very similar between the vertical (0°) and oblique (30°) load. In the Br configuration, the middle implant is missing, meaning that the force is only distributed between the outer implants. Thus, the loads on these two supporting implants increased by 25% on average (21% in Br-30° and 35% in Br-0°), depending on the load condition. Like the Sp configuration, the load distribution between the vertical and tilted load was almost identical in most cases. But, when loading the middle unit of the prosthesis (pontic) vertically (Br-0°-2), the force effect on the mesial implant (2nd premolar) was higher. In contrast, the distal implant (2nd molar) was loaded the most under oblique loading.

### Local stress accumulation (von Mises analysis)

**Fig. 5:**
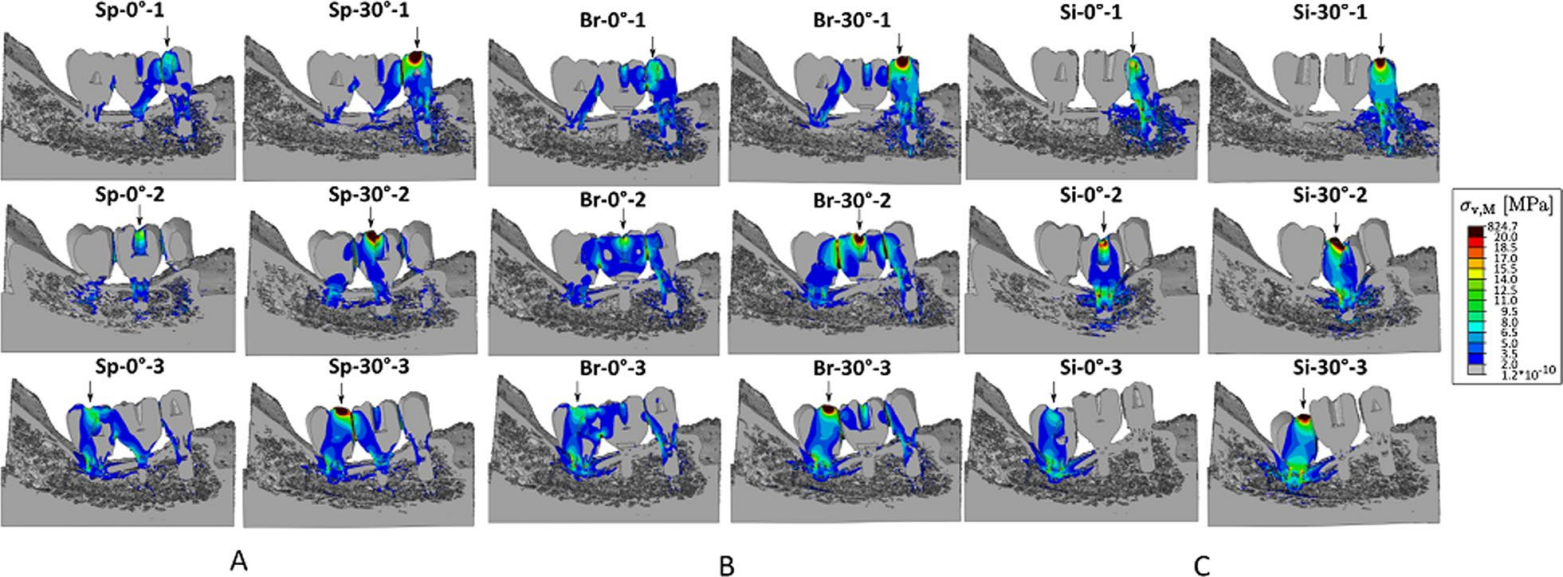
von Mises stress distribution for every load case of tested configurations. **A** Splinted crowns configurations. **B** Bridge configurations. **C** Single crowns configurations. Deformation scale factor: 600 ×. The yield stress limit of the bone is equivalent to stresses above 20 MPa (dark red) (100 N applied instead of physiological value of 500 N, rescale factor = 0.2 → σ_y_ = 100 MPa × 0.2 = 20 MPa)

The patterns of local stress accumulation in the implant/prosthesis/bone system are represented by von Mises stress maps in all test configurations (Fig. 5), compared to a physiological yield stress of 100 MPa for mandibular bone [28], because it is the only biological tissue considered in this study. Given that the yield strength of the remaining materials is a multiple of the bone yield limit [30], the bone is the only material that would be likely to permanently deform under the physiological loading conditions. The applied load was 100 N onto the indicated crown, according to the validation experiment. However, since this level of force is 5 times below the physiological load, which is assumed to be 500 N on average [31], a critical stress value of 100 MPa was adjusted with equidistant steps from 5 to 20 MPa. Thus, stresses above 20 MPa at maximum physiological loading would likely result in an in-elastic response of the bone and are marked in dark red, whereas stresses below 10% of the supposed yield limit are marked grey for a better visualisation. The blue areas correspond to stress values above 10% of the yield limit, which is equivalent to stresses above 10 MPa in a physiologic loading mode.

The comparative von Mises stress values above the yield limit (σ_v,M__>_ 20 MPa) measured in the bone tissue surrounding the implants are plotted in Fig. 3 (centre) to show the fraction of regions corresponding to dark red regions in Fig. 5. It is obvious that in all configurations there are elements located closed to the implant interface that are loaded beyond the yield strength, even in the bone region of the pontic in the Br configuration where the central implant is missing (Br-0°-2 and Br-30°-2), with the peri-implant tissue in the Si-2 configuration apparently exposed to the highest yield stress peaks.

In the 3-implant splinted configuration (Sp), local stresses above 10 MPa (equivalent) concentrated in the loaded crown and the underlying implant, but locally increased stresses were also observed at the crown/implant interface at remaining positions (Fig. 5A). Under oblique load (right column), local stresses were higher and distributed over a wider area with the local peaks concentrated at the occlusal surface of the loaded crown.

In the Br configurations local stresses were only concentrated around implant 1 and 3, as the middle implant was missing. Consequently, the elevated stresses were distributed over a larger area of the crowns and the implants (Fig. 5B). In addition, the local stresses within and around the load-bearing implants were higher than in the Sp configuration, especially in the extra-short implant with local load peaks of about 100 MPa (equivalent). Comparable to Sp configuration, oblique loading tended to increase the area and magnitude of elevated stresses.

In the Si configuration (single crowns), raised stresses were always located around the loaded crown, since no neighbouring contacts were modelled. The stress area was much larger than in splinted configuration. Furthermore, local stresses were partly above the physiological yield limit of bone (dark red) in all three implant positions. The overloaded areas under off-axis load were mainly concentrated at the occlusal surface of the crown (Fig. 5C).

### Strain energy density accumulation (bone tissue analysis)

**Fig. 6:**
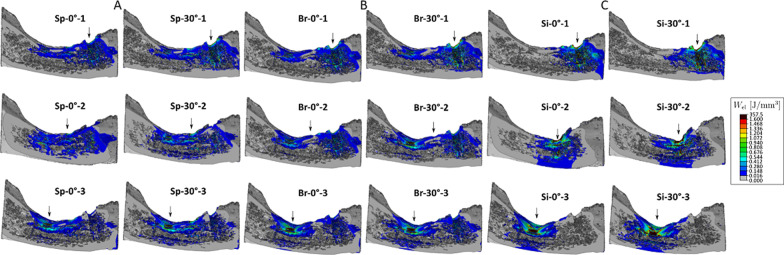
Strain energy density (SED) shown for every load case of tested configurations. **A** Splinted crowns configurations. **B** Bridge configurations. **C** Single crowns configurations. Deformation scale factor: 600 ×. Values above 200% of the optimal SED are marked dark and represent the regions of overload (above 1.6 J/mm^3^ (dark red); 100 N applied, instead of physiological value of 500 N, rescale factor = 0.2 × 0.2 = 0.04 → SED_opt_ = 20 J/mm^3^ × 0.2 × 0.2 = 0.8 J/mm^3^)

The tissue loading, represented as strain energy density (SED) values, was evaluated to get insight into physiological energies present in bone tissue. Since the SED is defined for bone, all other components were hidden and only the mandible was displayed. The SED is optimised by remodelling to 20 J/mm^3^ in bone [29]. Values above 200% of the optimal SED were marked dark red, while the areas with the optimal SED were highlighted in green for all configurations (Fig. 6). Bone areas with values below 2% of the optimum SED were presented in grey.

The corresponding numerical values to the dark red regions (i.e., values above 200% of the optimum SED) are displayed in the bottom boxplots in Fig. 3 to demonstrate the fracture of overloaded regions only. It can be seen that the values of the non-physiological SEDs are greatest around the 4-mm implant in all load cases. Further, the splinted and bridged configuration shows similar results, whereas the single configuration displays larger overloaded regions for all implant lengths.

In the Sp configuration (Fig. 6A), the trabecular bone region around the 8-mm implant (Sp-0°-1 and Sp-30°-1) showed a SED below or close to optimal values in both loading conditions, whereas low SED values were also distributed around the unloaded implants. Loading of the middle unit of the splinted prostheses (Sp-0°-2 and Sp-30°-2) resulted in a similar distribution pattern of SED, but also the bone around the 4-mm implant exhibited an increased SED compared to load case Sp. The SED in this region reached the values of more than 200% above the optimum limit, when the load was applied on the crown of the 2nd molar (Sp-0°-3 and Sp-30°-3). Here, the implant was mainly connected to the cortex leading to a much higher local SED than in the other two configurations.

When occlusal load was transferred to only two implants (Br configuration), the SED was locally higher than in the Sp configuration (Fig. 6B). The bone around the extra-short implant was stressed above the optimum limit also when the force was applied on the pontic (Br-0°-2 and Br-30°-2).

The removal of the splinting between the crowns (Si configuration) resulted in the local SED being above the optimal limit in each load case and limited to the region around the loaded implant (Fig. 6C).

## Discussion

This numerical study simulated a common clinical situation of a posterior mandible after long-term tooth loss, where patients widely seek for fixed implant rehabilitation of the edentulous region. Often, in such cases, low residual bone volume does not allow the insertion of implants with a standard length of 10 mm or more, thus the placement of short implants is an alternative option to avoid more invasive surgeries such as bone grafting and lateralisation of the alveolar nerve. However, a reduced contact area between the implant and the bone leading to an unfavourable crown-to-implant (C/I) ratio (> 1:1) with regard to greater bending moments [32], may jeopardise the long-term biomechanical success of the implant rehabilitation, especially in areas of strong occlusal loading [33, 34]. In fact, stress accumulation and distribution patterns are affected by a wide scope of factors such as diameter, number and distribution of the implants, type of prosthetic connection, bone characteristics, prosthesis material and design, and occlusal adjustment [5, 35, 36] making the clinical decisions and the establishment of guidelines for the usage of short dental implants rather complex. In this context, the present study intended to verify the influence of different loading and implant/prosthesis configurations on the global stiffness and the local stresses around the short implants supporting 3-unit prosthesis by FEA using linear static methods. The analysis is limited to the linear-elastic region since the maximum stresses have to be below the yield stress levels to avoid damage of the bone/implant/prosthesis system.

Modelling different clinical scenarios by FEA to predict stress/strain distribution at peri-implant regions and to investigate the influences of biomechanical factors is one of the most used computer-aided analysis in implant dentistry. Dumont et al. [37] stressed the importance of experimental validation of FEA studies of biological structures to assess the modelling error. Similar to the validation approach reported by Rocha Ferreira et al. [30], the current FE-analysis acquired the experimental data for comparison with model predictions in the previous mechanical study [24], where all test configurations were investigated in quasi-static manner using an optical deformation tracking system (ARAMIS). The comparative analysis of the global stiffness showed that the FEA-stiffness is overestimated by a factor of 7.3 for pooled data. Consequently, absolute stress/strain values presented here have to be treated carefully. Nevertheless, the main purpose was to compare biomechanical performance of different configurations rather than report absolute values.

Besides, limitations regarding unrealistic simulation of the structure’s material properties and the distribution of masticatory forces as well as other simplifications must be considered. FEA of the current study has several simplifications: first, no contact was simulated between the interfaces and elements were simply perfectly bonded at the interface between bone and implants. As there are indeed contact regions between piston (i.e., load-application device used in the experiment)–ball (used in the experiment to maintain a reliable contact surface in vertical loading)–prosthesis–abutment screw–implant–bone–embedding–aluminium block, a much lower experimental stiffness is reasonable. In truth, the mandible is comparable to a cantilevered bending beam, which accordingly transfers the forces acting on the bone. In the experiment/simulation, however, the pattern of stress distribution is affected by the embedding, so that the findings here primarily reflect the biomechanical behaviour of the implant. Given the rigid bond of all elements of the model, the influence of the thread width, which varies between the different implant lengths, is also much smaller in the simulation than in reality. In fact, no local stress peaks were observed since the implant is much stiffer than the bone. Further, adaptive meshing was used to model smaller parts more accurately and hence, the elements in the thread are somewhat smaller than the average element length of 0.1 mm (see Fig. 2). Furthermore, the masticatory conditions within the virtual environment of the study are not able to reproduce all details of the complex and varying bite forces. Still, the loading conditions applied in our study considered the axial and lateral components of the total bite force, even though only single point forces were used.

In contrast to numerous FE-studies on stress distribution in peri-implant bone tissue, which used a simplified bone geometry [15, 34–36, 38–40], our FE-models were derived from micro-CT images representing the actual specimen-specific architecture of the cortical and trabecular bone. Although using a single bone sample, this method of bone modelling allows a highly accurate characterisation of the external bone geometry and its internal microarchitecture [41] by conversion of the micro-CT image into voxel-based finite element model. Each element is assigned the same elastic modulus. However, possible anisotropy as well as variations in bone density remains the error sources. The implant lengths were selected based on the anatomical location, by using longer implants in regions where more bone was present (position 1), and shorter ones where only the cortex was giving support (position 3). As an extension, the current FE-model might allow in future usage of a parameterisation to virtually place different implants (in length and diameter) at the same anatomical location, to directly evaluate the best fitting implant, based on stress and SED.

In this study, 3 different prosthesis/implant configurations were evaluated under axial and oblique loads when the second premolar, first and second molar in a highly atrophied mandible are missing. The focus was on a global (structure) stiffness analysis to describe the displacement of the elements in response to the applied forces, and on VMS (i.e., von Mises stress) and SED (i.e., strain energy density) distributions within the individual components of the implant/prostheses/bone system and the bone tissue, respectively. The VMS are shearing stresses in the areas where plastic strains might take place [42], whereas the SED indicates the strains often correlated with bone remodelling activities [43–45]. The results demonstrated that the implant loading (i.e., forces on the implants) and subsequent bone stress/strain distribution were affected by the test parameters. Thus, the initial hypotheses of the study were rejected.

Regarding the splinting factor, the main results showed that splinting adjacent short implants is essential for load distribution between the individual implants and for reducing peri-implant stresses. In single-crown configurations, stresses concentrated around the loaded implant, leading eventually to easier biomechanical failure compared to splinted implants. This finding is consistent with clinical observations and other in vitro simulation studies [46–48], which suggested the biomechanical advantage of splinting short implants in the posterior mandible. In addition, the effect of splinting on load transfer seems to be particularly important under oblique forces, as they increased the magnitude and distribution of local stresses in all test configurations (Fig. 5A–C), similar to what has been reported in other studies using FEA [34, 36, 49]. However, a rationale of splinting implants by prosthetic components to favourably distribute the off-axis loads and to prevent the transfer of damaging force levels to the restoration and the supporting bone [50], might be questionable when using regular length implants. Several authors [51, 52] doubted the benefit of load sharing by splinted crowns maintaining that non-splinted restorations allow an optimal stress distribution to the supporting implants of regular length. Toniollo et al. [48] pointed out that splinting of regular and short implants in same context can overload the surrounding bone of the longest implant. Hence, the beneficial effect of splinting increases with the reduction of the implant length. A possible explanation could be due to the increase of the crown-to-implant ratio caused by reduced implant lengths (Table 1), which subsequently enhances the lever action and generates higher force moments [53]. Thus, increasing the total load area by splinting the short implants promotes a resistance of the bone against occlusal loading.

Comparing the global stiffness of the investigated models (Fig. 3 top), the splinting of the implants also revealed an obvious stiffening effect by a factor of 1.5 versus single crowns. Hereby, the 3-implant splinted configuration was 21% stiffer than the implant-bridge design on 2 implants. The largest stiffness (41 kN/mm) was observed at vertical loading of the middle implant (6 mm), most likely because the force in this loading position is distributed almost evenly over all three implants. The vertical load on the extra-short implant (4 mm) of the splinted configuration reduced the stiffness by 33%. This could be related to the fact that this implant was mainly connected to the cortical bone and there was almost no cancellous bone underneath to support the load and to contribute to a better force transmission. Interestingly, the off-axis loading resulted in a decreased stiffness of all test configurations (33–39% less for the splinted, and 30–37% less for the single crowns). It should be noted, however, that the single crowns were modelled with no interproximal contact; therefore, under clinical conditions, the non-splinted restorations would be a little bit stiffer depending on the interproximal contact tightness. Nevertheless, increased contact tightness has been proved to raise the stress intensity along the loaded implant [14].

The force distribution maps of splinted configurations (Fig. 4) showed that forces transferred to the neck region of the supporting implants, are very similarly distributed in the vertical and tilted configurations. When the middle implant is missing, the force is only distributed between the outer 2 implants resulting in an increased load on each implant by 25% (on average), compared to the 3-implant splinted configuration. Consequently, the residual effect of the stresses transmitted to the supporting structures (displayed as VMS) was higher in the 2-implant case, especially in the extra-short implant (Fig. 5B). Moreover, in this configuration, loading each implant resulted in locally increased SED of the peri-implant region of the loaded implant, which appears to be indicative of overloaded areas. Pronounced peri-implant bone strains were also noticed in response to occlusal forces acting on the pontic of the implant bridge (Fig. 6B). Christen et al. [44] concluded that local tissue loading activates sites of bone remodelling linearly dependent within a physiological range of bone loading. For this reason, an increased bone remodelling activity is likely in 2-implant configurations. Accordingly, the increased number of implants acts favourably in load sharing and the transmission of force to the splinted implants and helps to control the bone strain within a physiologic limit.

It is understood that the transmission of forces arising from functional or parafunctional (e.g., bruxism) loading will occur at bone/implant interfaces. Thus, one can assume that the smaller the region of bone contact, the higher the risk of overload [54]. Bourauel et al. [55] actually observed that peri-implant stresses/strains around short implants were markedly increased compared to those in standard implants. The same was confirmed in another study on short implants [56]. The effect of the short implant length, in our study, is best noticeable in single-crown configurations, as there is no splinting to absorb the increased stress concentrations and the stiffness is remarkably lower (Fig. 3 top). Here, not only a larger area around the implant was exposed to elevated stresses, but also more individual elements were overloaded (Fig. 3 centre; Fig. 5C). The same applies for the SED values compared to splinted configurations; the local elevated SED region was remarkably bigger (Fig. 6C). It was also apparent that the level of stresses and strains around the extra-short implant (4 mm) was higher compared to the other two implants, as was the fraction of elements over 200% of the optimum SED (Fig. 3 bottom). This suggests that when using short and extra-short implants in a non-splinted design, an increased bone remodelling is expected resulting potentially in more bone deposition (i.e., osseointegration) [44]. However, it is widely believed that micro-cracks, occurring at regions with high tissue loading trigger bone resorption to replace damaged tissue [57–59]. In this sense, the SED magnitudes around short and extra-short implants can also imply a higher risk of bone loss and fatigue of implant components upon overloading (i.e., biomechanical failure).

Taken together, this FEA study demonstrated the biomechanical interactions of short implant (4 mm, 6 mm, and 8 mm)—supported fixed partial dentures in the posterior mandible with variation in number of implants and prosthetic protocols in a qualitative way. However, for the treatment planning of a real situation, various other clinical parameters (e.g., implant offset, angulations, diameter, abutment height, inter-arch space) and individual factors (e.g., bone quality, bone atrophy level, parafunctional habits) which are beyond the scope of the current work must be regarded. In this sense, future research should be devoted to the development of parametrised models to predict the biomechanical conditions of an intended treatment based on the individual parameters of the patient. Finally, great efforts must be made to overcome the shortcomings of the numerical stress simulation studies in terms of the accuracy of absolute readings.

## Conclusions

Within the limitations of this FE-study, we concluded that the splinting of adjacent short dental implants by the prosthetic construction has a profound effect on the magnitude and distribution of the local stress peaks in peri-implant regions. Besides, replacement of each missing tooth by 1 implant is recommended, whenever bone supply and costs permit. Finally, it was shown that the prosthetic design (splinted versus non-splinted crowns) as well as the different implant lengths and loading directions affect the stress distribution and local stress accumulation in implant/prosthesis systems and surrounding bone, thus allowing the rejection of the research hypotheses.

## Data Availability

The datasets used and/or analysed during the current study are available from the corresponding author on reasonable request.

## Abbreviations

CAD/CAM: Computer-aided design/computer-aided manufacturing
3-D: Three-dimensional
FE: Finite element
IL: Implant length
SED: Strain energy density
FPD: Fixed partial denture
FEA: Finite element analysis
CH: Crown height
C/I: Crown-to-implant ratio
µCT: Microtomography
PMMA: Polymethyl methacrylate
CGAL: Computational geometry algorithms library
DOF: Degree of freedom
Sp: Splinted crowns
Br: Dental bridge
Si: Single crowns
VMS: Von Mises stress
